# Genomic Signatures of Artificial Selection Underlying Oil Content Differentiation in Chinese and Uruguayan Soybean Germplasm

**DOI:** 10.3390/plants15050800

**Published:** 2026-03-05

**Authors:** Xin Su, Huilong Hong, Yuehan Chen, Xiang Zhang, Mingxuan Gong, Jhon Larzábal, Juan E. Rosas, Jun Wang, Zhengwei Zhang, Yongzhe Gu, Lijuan Qiu

**Affiliations:** 1 The Shennong Laboratory/Institute of Crop Molecular Breeding, Henan Academy of Agricultural Sciences, Zhengzhou 450002, China; 18746428101@163.com (X.S.);; 2 College of Agronomy, Henan Agricultural University, Zhengzhou 450002, China; 3 The National Key Facility for Crop Gene Resources and Genetic Improvement (NFCRI)/State Key Laboratory of Crop Gene Resources and Breeding/Key Laboratory of Crop Gene Resource and Germplasm Enhancement (MOA)/Key Laboratory of Grain Crop Genetic Resources Evaluation and Utilization, Institute of Crop Sciences, Chinese Academy of Agricultural Sciences, Beijing 100081, China; honghuilong@caas.cn (H.H.); yuehan_c@163.com (Y.C.);; 4 College of Agronomy, Shenyang Agricultural University, Shenyang 110866, China; 5 College of Life Science and Technology, Harbin Normal University, Harbin 150025, China; 6 College of Agriculture, Northeast Agricultural University, Harbin 150030, China; 7 Agricultural and Livestock System, Instituto Nacional de Investigación Agropecuaria (INIA), Estación Experimental INIA La Estanzuela, Colonia CP70000, Uruguay; 8 Plant Breeding and Biotechnology Area, Instituto Nacional de Investigación Agropecuaria (INIA), Estación Experimental INIA Las Brujas, Canelones CP90100, Uruguay; jrosas@inia.org.uy

**Keywords:** soybean, oil content, population genomics, selective sweep, *GmDGAT1*

## Abstract

Soybean is a primary global vegetable oil source, yet modern South American cultivars often exhibit superior oil content compared to those from China, the center of origin. Elucidating the genetic basis of this differentiation is crucial for enhancing production efficiency. In this study, we systematically evaluated 98 representative accessions, comprising Chinese germplasm (CN) and Uruguayan germplasm. The latter included Uruguayan conventional germplasm (UY_N, where ‘N’ indicates ‘Normal’, meaning non-transgenic) and Uruguayan transgenic germplasm (UY_T). Using the “Zhongdouxin No. 1” SNP array and multi-environment phenotypic data. Uruguayan germplasm exhibited significantly higher mean oil content (21.48%) than Chinese germplasm (19.42%, *p* < 0.001), with high heritability (*H*^2^ ranging from 0.78 to 0.92). Genetic analysis revealed significant differentiation (mean *F_ST_* = 0.14), with Uruguayan lines showing reduced diversity due to breeding bottlenecks. Genome-wide scans identified differentiation in genomic regions harboring known lipid biosynthesis genes; notably, the high-oil allele frequency of *GmDGAT1* was 78.3% in Uruguayan germplasm versus 25.7% in Chinese lines, and the favorable *GmbZIP123* haplotype was fixed in the Uruguayan population. Uruguayan accessions also carried significantly more favorable alleles (18.3) than Chinese accessions (14.8). We conclude that high-oil traits in Uruguayan soybean result from the systematic stacking of favorable haplotypes at key loci via directional selection. Consequently, we propose incorporating South American high-oil allelic modules into the broadly adapted genetic backgrounds of Chinese cultivars to bridge the oil content gap.

## 1. Introduction

Soybean (*Glycine max*) stands as one of the most economically significant crops globally, providing approximately 28% of the world’s vegetable oil and 70% of its dietary protein [1,2]. Archeological and genomic evidence indicates that cultivated soybean originated in China, having been domesticated from wild soybean (*Glycine soja*) approximately 5000 years ago [3,4]. With the rapid rise of the global bioenergy industry and the continuous escalation in demand for edible oil, enhancing seed oil content has emerged as a central objective in modern soybean breeding [5,6].

Although China possesses the world’s most extensive collection of wild soybean and landraces [7], the primary production regions and breeding centers for soybean have shifted to the Americas—specifically the United States, Brazil, Argentina, and Uruguay—over the past half-century [8,9]. Long-term geographic isolation and heterogeneous breeding objectives have resulted in significant differentiation in agronomic traits between Chinese and foreign germplasm [10,11]. Historically, influenced by dietary culture, Chinese breeding has prioritized high protein content and quality suitable for soy food processing [12]. In contrast, breeding systems in the Americas primarily serve the industrial oil extraction and feed meal production sectors, driving a strong selection for high yield and high oil extraction rates [12,13]. This disparity has led to a notable divergence in oil content, with Chinese varieties generally exhibiting 2–3 percentage points lower oil content compared to commercial varieties from the Americas [14].

Despite its origin in China, soybean has undergone extensive adaptation in South America. A recent study characterized the genetic diversity and grain traits of Chinese and Uruguayan soybean germplasm, revealing significant phenotypic and genetic differentiation [15]. However, the genomic signatures of artificial selection specifically underlying oil content differentiation between these two populations remain to be elucidated. The biosynthesis of soybean seed oil primarily proceeds via the glycerol-3-phosphate pathway [16], involving the synergistic action of a series of key enzymes, such as diacylglycerol acyltransferase (DGAT) and glycerol-3-phosphate acyltransferase (GPAT) [17,18]. Among these enzymes, *GmDGAT1* catalyzes the final step of triacylglycerol (TAG) synthesis and is considered a rate-limiting enzyme for oil accumulation [19,20,21]. Furthermore, transcription factors such as *GmWRI1* [22,23], *GmMYB73* [24], and *GmbZIP123* [25] play critical roles in oil accumulation by regulating the lipid synthesis network. Artificial selection, as the core driver of crop improvement, leaves “selective sweep” signatures on the genome, characterized by reduced diversity and high population differentiation indices (*F_ST_*) [26,27,28].

Currently, systematic analysis of germplasm resources from Uruguay—an emerging soybean powerhouse in South America—remains insufficient. In particular, the genetic basis and key regulatory loci underlying their high-oil phenotypes have not been fully elucidated [29,30,31]. Leveraging the resources from the China–Uruguay Joint Laboratory for Soybean Research and Innovation, established under the “Belt and Road” initiative, this study introduces 26 novel transgenic accessions and 24 novel conventional accessions from Uruguay, alongside 48 elite accessions exported to Uruguay. We utilized the laboratory-developed “Zhongdouxin No. 1” genotyping array to identify these 98 accessions, combining high-density SNP genotyping with multi-environment phenotypic data.

This study aims to address the following scientific questions: (1) Clarify the degree of phenotypic differentiation and genetic stability regarding oil content between Chinese and Uruguayan germplasm; (2) Reveal genome-wide artificial selection footprints within Uruguayan germplasm; and (3) Elucidate the haplotype structure of key genes and the pyramiding mechanism of multi-locus favorable alleles. The results of this study will not only facilitate the dissection of the genetic mechanisms underlying high-oil traits in soybean but also provide a solid theoretical foundation and core germplasm support for the collaborative innovation and utilization of Sino–Uruguayan soybean resources and the molecular design breeding of new high-oil varieties.

## 2. Results

### 2.1. Variation in Seed Protein and Oil Content Among Chinese and Uruguayan Soybean Populations

**Figure 1:**
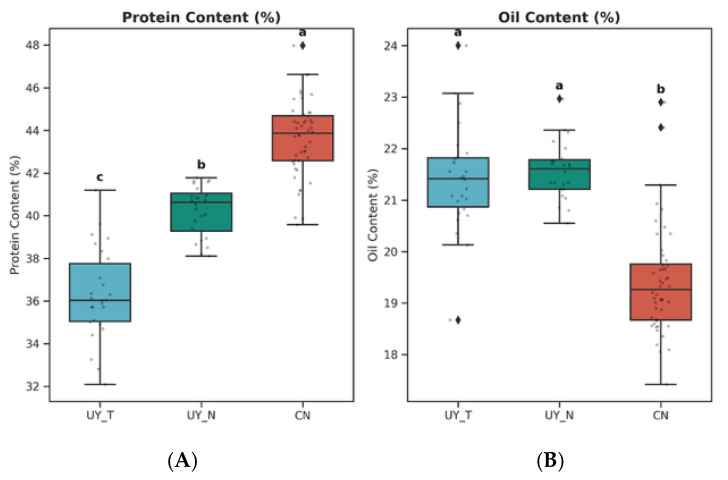
Phenotypic variation of seed quality traits in Chinese and Uruguayan soybean germplasm. (**A**) Boxplot of protein content (%). (**B**) Boxplot of oil content (%). The centerline in each box represents the median, the edges represent the upper and lower quartiles, and the whiskers indicate the range of non-outlier data. Different lowercase letters indicate significant differences among groups (*p* < 0.05, one-way ANOVA followed by Tukey’s HSD post-hoc test). CN, Chinese germplasm; UY_N, Uruguayan conventional germplasm (where ‘N’ denotes ‘Normal’, indicating non-transgenic status); UY_T, Uruguayan transgenic germplasm.

To evaluate the variation in seed quality traits across different germplasm resources, we analyzed the protein and oil content of three distinct soybean populations: Chinese (CN), Uruguayan Conventional (UY_N), and Uruguayan Transgenic (UY_T). The descriptive statistics and distribution of these traits are presented in Figure 1.

Significant genotypic differences were observed in seed protein content among the three populations (*p* < 0.05) (Table 1). The CN population exhibited the highest protein content, with a mean value of 43.61% (±1.18), which was significantly higher than both Uruguayan populations. The UY_N population followed with a mean protein content of 40.24% (±1.18). The UY_T population showed the lowest protein content at 36.30% (±2.14). Statistical analysis (Tukey’s HSD) confirmed that the protein levels differed significantly between all three groups (CN > UY_N > UY_T), as indicated by the distinct significance letters (a, b, and c) in Figure 1A.

Conversely, the oil content demonstrated a contrasting trend, consistent with the well-known negative correlation between protein and oil accumulation in soybean seeds. The two Uruguayan populations (UY_N and UY_T) displayed significantly higher oil content compared to the Chinese germplasm. The UY_N and UY_T populations had mean oil contents of 21.56% (±0.55) and 21.41% (±1.03), respectively. Statistical analysis revealed no significant difference between the conventional and transgenic Uruguayan lines. However, both were significantly higher (*p* < 0.05) than the CN population, which averaged a 19.42% (±1.05) oil content.

### 2.2. Broad-Sense Heritability Analysis of Oil Content

Broad-sense heritability (*H*^2^) analysis was conducted across three distinct groups: the Chinese (CN), Uruguayan conventional (UY_N), and Uruguayan transgenic (UY_T) populations (Table 2). The results revealed high heritability estimates of 0.87 for the CN population, 0.82 for the UY_N population, and 0.98 for the UY_T population, indicating strong genetic stability across diverse environments. To further assess the potential for genetic gain in conventional cross-breeding, we analyzed a “Combined” population consisting of the 72 conventional accessions (pooling 48 CN and 24 UY_N). This combined analysis revealed an increased broad-sense heritability of 0.92.

A comprehensive analysis of genetic stability combining both Chinese and Uruguayan germplasm revealed an increased broad-sense heritability of 0.92. The increased broad-sense heritability (*H*^2^ = 0.92) in the combined population reflects the substantial genetic divergence regarding oil traits between the two gene pools, indicating that the phenotypic differences are largely driven by fixable genetic factors rather than environmental plasticity. This elevation is primarily attributed to the significant expansion of the range of genetic variation resulting from the integration of the two distinct germplasm pools. Specifically, the genotypic mean square (*MS_G_*) increased markedly from the individual population values (2.12–5.56) to 11.97 in the combined analysis, thereby increasing the proportion of genetic variance within the total phenotypic variance. These results confirm that oil content is a stable trait with high heritability. Consequently, hybridization between Chinese and Uruguayan germplasm holds significant promise for generating high-oil progeny with exceptional genetic stability, thereby enriching the genetic resources available for improving the soybean oil content.

### 2.3. Phenotypic Divergence in Oil and Protein Content Between Chinese and Uruguayan Soybean Germplasm

**Figure 2:**
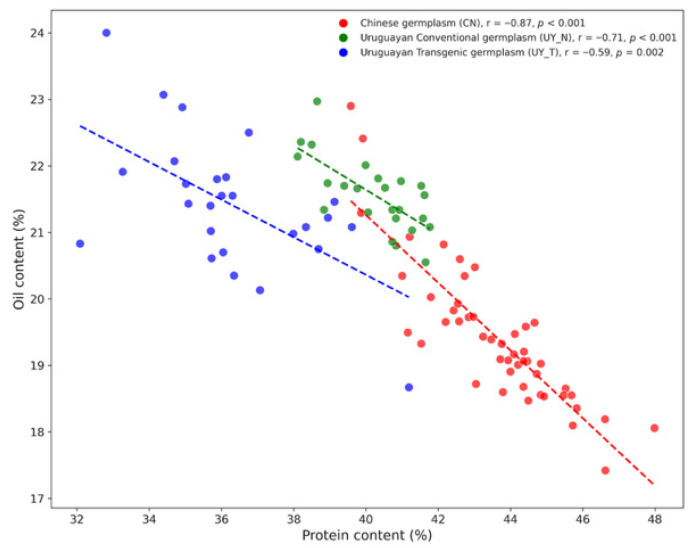
Pearson correlation analysis between seed protein and oil content in Chinese and Uruguayan soybean populations. The scatter plot displays the relationship between protein content (%) on the x-axis and oil content (%) on the y-axis. Red: Chinese germplasm (CN). Green: Uruguayan Conventional germplasm (UY_N). Blue: Uruguayan Transgenic germplasm (UY_T). Dashed lines indicate the linear regression trends between protein and oil content for each germplasm group, with correlation coefficients (*r*) and *p*-values.

Correlation analysis was performed on Chinese conventional germplasm, as well as Uruguayan conventional and transgenic germplasm, across multiple environments (including Hainan, Zhejiang, and Jiangxi). The results revealed a highly significant negative correlation between seed protein and oil content in both Chinese and Uruguayan populations (Figure 2). The Chinese germplasm was predominantly distributed in the lower-right quadrant of the scatter plot, characterized by a “high-protein (mean 43.6%), low-oil (mean 19.4%)” phenotype. In contrast, the Uruguayan conventional and transgenic germplasm clustered in the upper-left quadrant, exhibiting a distinct “high-oil (mean > 21.4%), medium-to-low protein (mean < 40.3%)” profile. This significant negative correlation, combined with the clear distributional divergence between the populations, further confirms the unique complementary advantages of Uruguayan germplasm for enhancing the oil content of Chinese soybean varieties.

### 2.4. Phenotypic Differentiation of Oil and Protein Content Between Chinese and Uruguayan Soybean Germplasm

**Figure 3:**
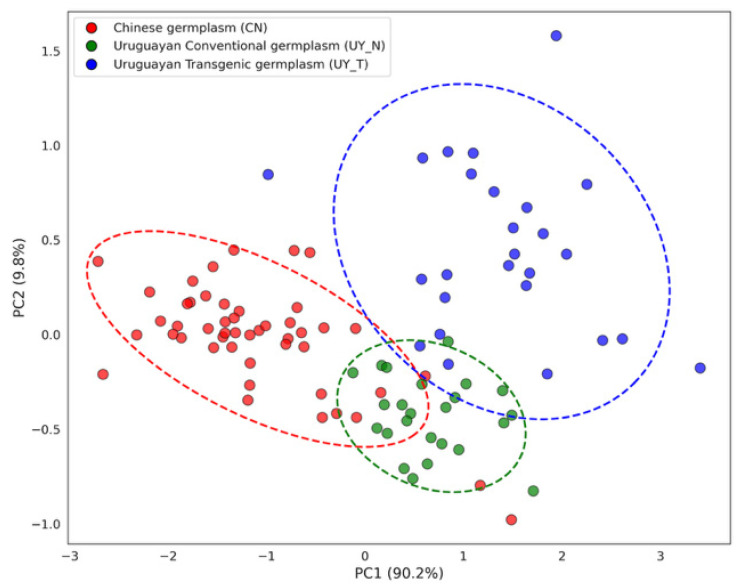
Principal component analysis (PCA) of seed quality traits in Chinese and Uruguayan soybean populations. The scatter plot illustrates the distribution of individual accessions based on the first two principal components (PC1 and PC2). Red dots: Chinese germplasm (CN). Green dots: Uruguayan Conventional germplasm (UY_N). Blue dots: Uruguayan Transgenic germplasm (UY_T). Dashed cycles indicate 95% confidence regions for each group.

To visualize the overall phenotypic structure and assess the divergence among the three soybean populations, Principal Component Analysis (PCA) was performed based on seed protein and oil content data (Figure 3).

PC1 accounted for the vast majority of the variation (90.23%) and primarily reflects the strong negative correlation between protein and oil content. As shown in Figure 2, there was a distinct separation along the PC1 axis between the Chinese germplasm (CN) and the Uruguayan populations (UY_N and UY_T). The CN population (marked in red) clustered loosely on the negative side of PC1 (driven by high protein/low oil), whereas the Uruguayan populations were concentrated on the positive side (driven by high oil/low protein).

PC2 explained the remaining 9.77% of the variance. Notably, the UY_T and UY_N populations exhibited a high degree of overlap and tight clustering. This spatial distribution suggests that the genetic background shared by the Uruguayan lines is the primary determinant of seed quality traits, and the introgression of transgenic events did not result in significant phenotypic divergence from the conventional recurrent parents regarding protein and oil composition. Conversely, the distinct spatial isolation of the CN population highlights the significant genetic distance and divergent breeding selection history between Chinese and South American germplasm.

### 2.5. Genome-Wide Distribution of Genetic Diversity in Chinese and Uruguayan Germplasm

**Figure 4:**
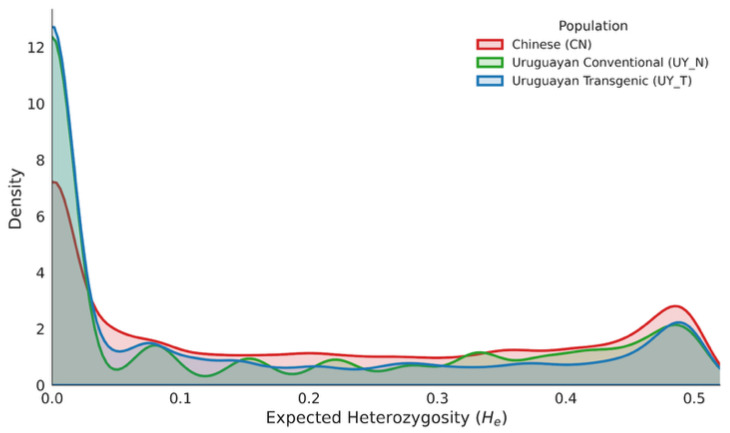
Genome-wide distribution of expected heterozygosity (*He*) in Chinese and Uruguayan soybean populations. The Kernel Density Estimation (KDE) plot illustrates the frequency distribution of genetic diversity based on high-quality SNP markers across the genome. The red curve represents the Chinese germplasm (CN), which exhibited the broadest distribution and highest diversity (mean *He* = 0.198), reflecting its status as the center of origin. The green curve represents the Uruguayan Conventional germplasm (UY_N) (mean *He* = 0.151), and the blue curve represents the Uruguayan Transgenic germplasm (UY_T) (mean *He* = 0.135).

To elucidate the patterns of genetic variation and the impact of breeding history, we characterized the genome-wide distribution of expected heterozygosity (*He*) across the three populations based on 158,327 SNP markers (Figure 4). The Kernel Density Estimation (KDE) plot revealed significant stratification in diversity structure among the Chinese (CN), Uruguayan Conventional (UY_N), and Uruguayan Transgenic (UY_T) germplasms.

The Chinese germplasm exhibited the highest level of genetic diversity, with a mean *He* of 0.198. Its distribution curve was relatively broad and flat, indicating that a rich reservoir of allelic variation had been maintained in these foundation parents from the center of origin. In contrast, both Uruguayan populations displayed a marked reduction in genetic diversity, characterized by a skewed distribution dominated by fixed or nearly fixed alleles (low *He*). This pattern reflects the “founder effect” and genetic bottlenecks associated with the introduction of soybean to South America.

Notably, by differentiating the Uruguayan germplasm, we observed that the Uruguayan Transgenic population (UY_T) exhibited the lowest genetic diversity (mean *He* = 0.135), which was lower than that of the Uruguayan Conventional population (UY_N, mean *He* = 0.151). Although the overall genetic backgrounds of UY_N and UY_T were similar, the further reduction in diversity in UY_T suggests that the introgression of transgenic events (e.g., glyphosate tolerance) and the subsequent rigorous selection for commercial traits have imposed an additional genetic bottleneck, leading to the fixation of a larger proportion of genomic loci.

### 2.6. Genome-Wide Selection Signals and Analysis of the Major Oil-QTL GmDGAT1 in Chinese and Uruguayan Germplasm

**Figure 5:**
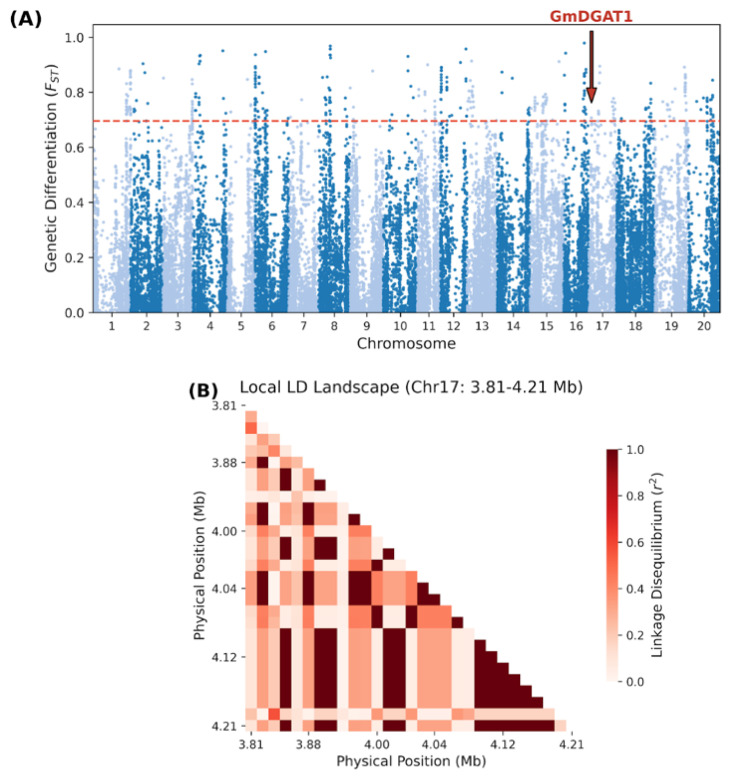
Genome-wide selection signals and local LD analysis. (**A**) Manhattan plot of the fixation index (*F_ST_*) between Chinese (CN) and Uruguayan (UY) germplasm. The red dashed line indicates the significance threshold, and the red arrow highlights the significant peak associated with *GmDGAT1* on chromosome 17. (**B**) Local Linkage Disequilibrium (LD) heatmap surrounding the *GmDGAT1* locus (Chr17: 3.8–4.3 Mb) in the UY population. Red blocks represent high *r*^2^ values, indicating fixed haplotype blocks resulting from strong artificial selection.

To investigate genomic differentiation and selection signals associated with breeding history and adaptation, we conducted a genome-wide scan between Chinese and Uruguayan soybean germplasm using the fixation index (*F_ST_*). We defined candidate sweep regions as the top 1% of *F_ST_* windows. The distribution of *F_ST_* values across the 20 chromosomes revealed distinct patterns of genetic differentiation (Figure 5A). A highly significant selection signal associated with oil content was identified on chromosome 17 (*F_ST_* = 0.51) (Table 3). This peak physically encompasses *GmDGAT1* (*Glyma.17G053300*), a key gene encoding diacylglycerol acyltransferase, which is crucial for soybean oil synthesis. Further local Linkage Disequilibrium (LD) analysis of the surrounding region (Chr17: 3.8–4.3 Mb) revealed a significant selective sweep in the Uruguayan population. As shown in Figure 5B, the Uruguayan germplasm exhibited extensive high-*r*^2^ LD blocks within *GmDGAT1* and its flanking regions. This indicates that the region has undergone strong artificial selection, leading to the rapid fixation of elite haplotypes and a significant reduction in genetic diversity. These genomic-level findings support the hypothesis that *GmDGAT1* is a major contributor to the oil content differentiation between Chinese and Uruguayan germplasm. The results suggest that variants within this segment have been preserved as an integral haplotype module during long-term commercial breeding. Consequently, these loci or their associated LD blocks experienced differential selection pressures during the establishment or improvement of the Chinese and Uruguayan breeding pools, which likely accounts for the significant phenotypic divergence in oil quality between the two germplasm resources.

### 2.7. Haplotype Differentiation and Pyramiding of Elite Alleles for Key Oil and Adaptation Genes

To elucidate the genetic mechanisms driving oil content differentiation, we prioritized candidate genes based on a stringent three-tier strategy: (1) location within the top 1% of genomic regions showing significant genetic differentiation (*F_ST_*) between Chinese and Uruguayan populations; (2) functional annotation related to lipid biosynthesis or regulation; and (3) distinct allele frequency shifts consistent with the phenotypic divergence.

**Figure 6:**
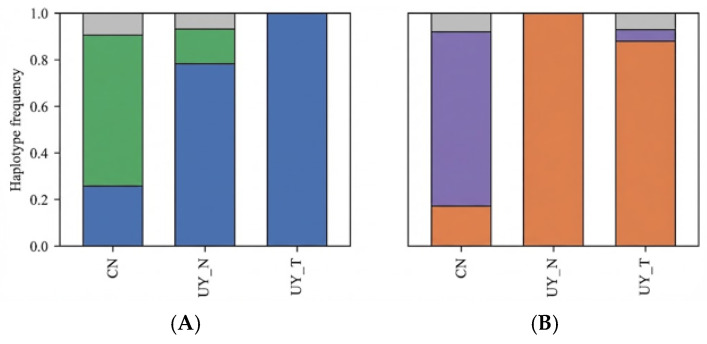
Frequency distribution of *GmDGAT1* and *GmbZIP123* haplotypes in different soybean germplasm. (**A**) Frequency distribution of haplotypes in the *GmDGAT1* gene region on Chromosome 17 across Chinese germplasm (CN), Uruguayan conventional varieties (UY_N), and Uruguayan transgenic varieties (UY_T). Blue: *GmDGAT1* Haplotype 1; Green: *GmDGAT1* Haplotype 2; Gray: *GmDGAT1* Haplotype 3. (**B**) Frequency distribution of haplotypes in the *GmbZIP123* gene region on Chromosome 06 across the three populations. Orange; *GmbZIP123* Haplotype 1; Purple: *GmbZIP123* Haplotype 2; Gray: *GmbZIP123* Haplotype 3.

Based on these criteria, two high-confidence candidate genes were selected for detailed haplotype analysis: *GmDGAT1* (*Glyma.17G130000*) and *GmbZIP123* (*Glyma.06G096700*). *GmDGAT1*, located in the most significant selection peak on chromosome 17, encodes diacylglycerol O-acyltransferase 1, a rate-limiting enzyme in the triacylglycerol (TAG) biosynthesis pathway. *GmbZIP123*, located on chromosome 6, encodes a basic leucine zipper transcription factor known to regulate genes involved in lipid transport and accumulation. Preliminary analysis indicated that favorable alleles at these loci are nearly fixed in the high-oil Uruguayan population but remain variable in the Chinese population, making them ideal targets to explain the observed phenotypic gap (Figure 6, Table 4 and Table 5).

At the *GmDGAT1* locus, three major haplotypes (Hap1–Hap3) were identified. In the Chinese germplasm, Hap2 was the dominant haplotype (frequency 0.650), while Hap1 appeared at a lower frequency (0.257). In contrast, the frequency of Hap1 significantly increased in Uruguayan conventional germplasm (0.783) and reached complete fixation in Uruguayan transgenic germplasm (1.000). This extreme shift in haplotype frequency suggests that the *GmDGAT1* region has likely undergone strong directional selection within the Uruguayan breeding population.

At the *GmbZIP123* locus, three major haplotypes (Hap4–Hap6) were identified. The Chinese germplasm was dominated by Hap5 (frequency 0.750), with a low frequency of Hap4 (0.170). In sharp contrast, Hap4 was completely fixed in Uruguayan conventional germplasm (1.000) and also predominated in Uruguayan transgenic germplasm (0.880). These results indicate an almost complete allele substitution at the *GmbZIP123* locus in Uruguayan germplasm, exhibiting typical characteristics of a selective sweep.

Integrating the haplotype distribution characteristics of both *GmDGAT1* and *GmbZIP123*, we observed a highly consistent trend of allele enrichment across multiple loci in the Uruguayan germplasm. This demonstrates that during long-term intensive breeding, Uruguayan germplasm has not only been directly selected for the high-oil gene *GmDGAT1* but has also likely fixed *GmbZIP123* alleles beneficial for enhanced metabolic adaptability. This mode of pyramiding “high-oil + high-adaptability” elite alleles constitutes an important genetic basis for their superior comprehensive agronomic traits and may contribute to Uruguayan high-oil soybean breeding.

### 2.8. Allelic Differentiation of Key Oil Genes Between Chinese and Uruguayan Populations

**Figure 7:**
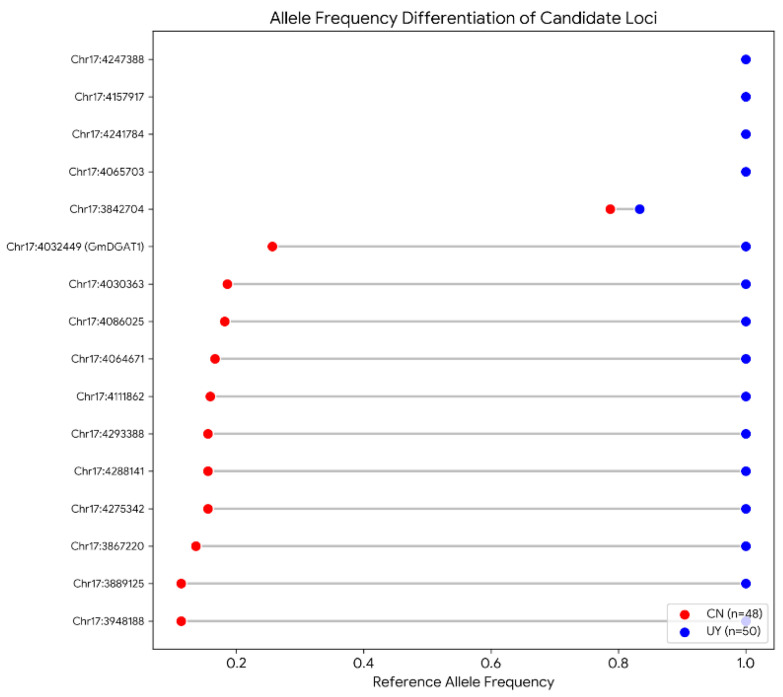
Allelic frequency differentiation of candidate oil biosynthesis genes between Chinese and Uruguayan soybean populations. The dumbbell plot illustrates the reference allele frequencies of key oil-related loci. Red dots represent allele frequencies in the Chinese germplasm (CN, *n* = 48), while blue dots represent the combined Uruguayan germplasm (UY, including conventional and transgenic materials, *n* = 50). Genes are ordered on the vertical axis based on the magnitude of frequency difference between the two populations, with the loci showing the greatest differentiation positioned at the bottom. The length of the gray connecting line indicates the degree of genetic differentiation at each locus.

Based on the statistics of 42 known oil-related gene loci compiled from the literature (Supplementary Table S1), we compared the allele frequencies of candidate genes involved in lipid biosynthesis and regulation between Chinese and Uruguayan soybean germplasms (Figure 7). As illustrated in the dumbbell plot, there was a significant variation in the degree of differentiation among different loci. Although some candidate loci exhibited relatively conservative frequency distributions between the two populations—suggesting that they may have undergone balancing selection or were not the primary targets of recent improvement in Uruguayan germplasm—other key loci displayed extremely significant frequency differences, indicating that these genomic regions have been subjected to intense differential selection pressure.

Most notably, *GmDGAT1*, a key determinant in the soybean triacylglycerol biosynthesis pathway, exhibited the most drastic frequency shift at its associated loci (e.g., Chr17:4032449). The frequency of the favorable reference allele at this locus was relatively low in Chinese foundation germplasm (~0.26) but approached fixation in the introduced Uruguayan germplasm (~0.90–1.00). This sharp contrast demonstrates that *GmDGAT1* underwent strong positive selection during the introduction of soybean to Uruguay or during local intensive high-oil breeding processes. This is likely a critical genetic factor contributing to the higher oil content observed in these elite germplasms. From a breeding perspective, this core SNP of *GmDGAT1* can serve as an efficient functional marker for direct application in marker-assisted selection (MAS) while also providing a clear target for future precision improvement of oil traits via gene editing.

## 3. Discussion

### 3.1. Evolution of Oil Traits and Genetic Differentiation Under Domestication Selection

This study quantified the substantial difference in oil content (~2.1%) between Chinese and Uruguayan soybean germplasms, confirming that significant genetic divergence has occurred in the emerging soybean production hubs of South America. This phenotypic differentiation profoundly reflects the distinct domestication and breeding histories of the two regions. Historically, Chinese soybean breeding has prioritized increasing protein content through artificial selection to meet the traditional demands for tofu and soymilk production, which has, to some extent, suppressed selection for high-oil traits [32]. Due to the significant negative correlation between protein and oil content in soybean seeds (approximately −0.6), this “evolutionary trade-off” driven by energy metabolic constraints means that selection for high protein is often accompanied by the retention of low-oil genes [14,33]. It is worth noting that while both Uruguayan populations exhibited lower protein content than the Chinese germplasm, the UY_T population was significantly lower (36.30%) than the UY_N population (40.24%). This likely reflects the “yield dilution effect” prevalent in elite commercial transgenic varieties, where intensive selection for high yield and oil extraction efficiency often exacerbates the reduction in seed protein concentration due to the negative correlation between these traits.

Although the population size was moderate, the significant phenotypic divergence allowed for the effective detection of major selection signatures distinguishing the two germplasms. In contrast, as a key link in the South American soybean production chain, the Uruguayan breeding system is deeply influenced by the North American model, primarily driven by industrial oil extraction efficiency. The pursuit of high oil yield has disrupted the original protein–oil balance, leading to the rapid accumulation of high-oil genes. In this study, *F_ST_* data strongly support this view: the genetic differentiation of Uruguayan germplasm at oil-related loci. This indicates that the differentiation is the result of high-intensity directional artificial selection rather than random genetic drift. This aligns with the domestication bottleneck phenomenon observed in soybean pan-genome studies by Li et al. (2014), where the improvement of specific agronomic traits was accompanied by a sharp decline in genomic diversity [31]. It is important to note that the high genome-wide differentiation observed is not solely attributable to selection for oil content. The distinct demographic history of South American soybean, characterized by a ‘founder effect’ from a limited number of North American ancestors, has likely elevated the baseline *F_ST_* due to genetic drift [34]. However, the peak at Chr17 (*GmDGAT1*) significantly exceeds this genomic background (top 1% outlier), suggesting that directional selection for oil traits has acted upon this locus in addition to demographic forces.

### 3.2. Selection Signatures and Functional Evolution of GmDGAT1

In this study, we detected significant differentiation signals (*F_ST_* exceeding the threshold) on multiple chromosomes. Although the highest genome-wide differentiation peaks were located in other regions (non-oil traits)—which may be related to background differences in photoperiod adaptability (e.g., *E*-series flowering genes) or disease resistance between Chinese and Uruguayan germplasms—the *GmDGAT1* region on chromosome 17 showed specific and significant selection signals for the oil traits focused on in this study (*F_ST_* = 0.514, Top 1%). Previous studies have reported that *GmDGAT1* is a key rate-limiting enzyme catalyzing the final step of triacylglycerol biosynthesis [16]. The significant differentiation at this locus provides direct functional genomic evidence for the substantial phenotypic difference in oil content between Chinese and Uruguayan germplasms.

One of the most important findings of this study is that the *GmDGAT1* gene is approaching fixation (78.3%) in Uruguayan germplasm. Previous research indicates that *DGAT1* has undergone convergent selection during the domestication of various oil crops, such as rapeseed and maize [35]. In soybean, non-coding variations in the *GmDGAT1* promoter region have been confirmed to significantly regulate its expression level during early seed development, thereby determining the final oil accumulation [36].

Our results suggest that Uruguayan germplasm likely successfully fixed the high-oil haplotype of *GmDGAT1* by introducing North American high-oil parents. Due to long-term selection pressure for high oil, the high-oil haplotype of *GmDGAT1* has been systematically retained and enriched in the Uruguayan population. Conversely, the frequency of this favorable allele is relatively low in Chinese germplasm (25.7%). This explains the generally lower oil content in Chinese varieties and implies that this locus has not yet been subjected to sufficient positive selection pressure in the history of Chinese breeding. Furthermore, we identified novel fixed loci on Chr6 (*Glyma.06G010200*) and Chr8, which may contain lipid regulators that have not yet been fully characterized. For instance, the presence of multiple genes related to fatty acid transport near the Chr6 locus may represent a lipid synthesis regulatory mechanism unique to South American germplasm [21]. While previous studies have characterized the enzymatic function of *GmDGAT1* [19,20,21], this study focused on genomic selection signatures. Future functional validation, such as expression analysis or transgenic complementation, is necessary to quantify the specific effect of the ‘Uruguayan’ haplotype on lipid accumulation efficiency compared to the ‘Chinese’ haplotype.

### 3.3. Diversity Loss and Genetic Gain Under Genetic Bottleneck Effects

Although Uruguayan germplasm exhibits an extremely high oil content, its genetic diversity is significantly reduced (Figure 3). This “founder effect”, derived from a few elite parents, is prevalent in American soybeans [34,37]. The loss of genetic diversity, known as “genetic drag”, while fixing superior traits, may also increase crop vulnerability to extreme climates [38]. In contrast, Chinese germplasm retains a richer reservoir of variation, providing a valuable genetic buffer for coping with future climate change.

Our study found minimal genetic difference between Uruguayan conventional and transgenic germplasms, suggesting that local transgenic breeding has primarily involved introducing resistance genes into a few elite high-oil genetic backgrounds (chassis). Conversely, the rich variation reservoir retained in Chinese germplasm offers valuable genetic resources for addressing future climate change and pest outbreaks. Therefore, the utilization of Uruguayan germplasm should extend beyond simplistic direct introduction. Instead, it requires precise genomic introgression to introduce high-oil gene modules while maintaining the broad adaptability of Chinese germplasm. We observed minimal genetic differentiation between Uruguayan conventional and transgenic germplasms. However, we acknowledge that this observation may be influenced by the sample size (*n* = 50) and the common practice of introgressing transgenes into established elite backgrounds (chassis varieties), which limits genetic diversity.

It is worth noting that while metrics such as nucleotide diversity and admixture proportions offer additional layers of population insight, the expected heterozygosity (*He*) utilized in this study serves as a robust proxy for genetic diversity in self-pollinating crops like soybean. Given that the germplasm assessed consists of established cultivars with high homozygosity, *He* effectively captures the allelic richness differences between the populations. Furthermore, the clear population stratification revealed by PCA (Figure 3) and the high average *F_ST_* values provide strong evidence of the distinct genetic backgrounds and limited recent gene flow between the Chinese and Uruguayan breeding pools, supporting the presence of independent selection histories.

### 3.4. Molecular Breeding and Germplasm Improvement Strategies Based on Genomic Differentiation

Based on the significant genetic differentiation regions and key fixed alleles identified between Chinese and Uruguayan soybean germplasm, we propose integrated molecular breeding strategies to enhance the oil content of Chinese soybeans.

First, future efforts should prioritize Precise Marker-Assisted Selection (MAS). Developing high-throughput KASP or SNP haplotype markers for regions such as Chr17 (*GmDGAT1*) will facilitate the efficient selection of individuals carrying elite Uruguayan alleles. Given the strong negative correlation between oil and protein content, Genomic Selection (GS) models should be employed to break the linkage drag between high-oil and low-protein genes.

Furthermore, Gene Editing technologies offer a direct pathway for improvement. Since *GmDGAT1* has undergone strong artificial selection, using CRISPR/Cas9 to modify cis-regulatory elements in Chinese cultivars to mimic the high-oil haplotype regulation could rapidly enhance the oil content. This approach should be complemented by the broad-spectrum excavation of wild gene resources, utilizing pan-genome analysis to mine rare alleles lost during domestication. Future work could explore gene editing technologies. Given the strong selection signal at *GmDGAT1*, this locus represents a potential target for future functional studies using CRISPR/Cas9 to investigate whether modifying cis-regulatory elements can recapitulate the high-oil phenotype in Chinese backgrounds.

In summary, Uruguayan germplasm resources represent an important gene pool for broadening the genetic background of Chinese soybean and improving oil traits. Conversely, Chinese germplasm plays a crucial role in enriching the high-protein characteristics of Uruguayan soybean. Integrating comparative genomics analysis with modern molecular breeding systems will not only help elucidate the genetic nature of oil differences between Chinese and foreign germplasm but also provide theoretical grounds and core germplasm support for new germplasm innovation in both countries.

## 4. Materials and Methods

### 4.1. Plant Materials and Phenotypic Investigation

A representative core collection consisting of 98 soybean germplasm accessions was constructed for this study. This collection was rigorously screened to maximize the coverage of genetic diversity in soybean breeding from both China and Uruguay. It included 48 backbone parents representing the export-grade Chinese soybeans (CN) and 50 core commercial varieties representing the mainstay of current agricultural production in Uruguay (comprising 24 conventional accessions, UY_N, and the Uruguayan transgenic accessions (UY_T) consisting of commercial glyphosate-tolerant varieties). This sampling strategy, based on a core collection, ensured the comprehensive capture of key genetic differentiation and elite allelic variations between Chinese and Uruguayan germplasms, even with a limited sample size. The field experiments were conducted using a Randomized Complete Block Design (RCBD) with two replications at each of the three locations (Hainan, Zhejiang, and Jiangxi). Each plot consisted of four rows, 3 m long, with a row spacing of 0.5 m. Prior to analysis, outliers were identified and removed using the Interquartile Range (IQR) method. The normality of residuals and homogeneity of variance were verified using Shapiro–Wilk and Levene’s tests, respectively

Broad-sense heritability (*H*^2^) was estimated using a linear mixed model (LMM) in the R package lme4. The model was fitted as *Y_ijk_* = *μ* + *G_i_* + *E_j_* + (*GE*)*_ij_* + *R_k(j)_* + *ε_ijk_*, where *G_i_* represents the random effect of the *i*-th genotype, *E_j_* represents the fixed effect of the *j*-th environment, *(GE)_ij_* is the genotype × environment interaction, and *R_k(j)_* is the effect of the *k*-th replicate nested within the *j*-th environment. Heritability was calculated as *H*^2^ = *σ*^2^*_g_*/(*σ*^2^*_g_* +*σ*^2^*_ge_*/*n* + *σ*^2^*_ε_/nr*), where *n* is the number of environments and *r* is the number of replications.

All materials were planted in multiple environments in Hainan, Zhejiang, and Jiangxi, China, during 2021–2022. After harvest, the crude fat and crude protein contents of the seeds were measured using a DA 7250 near-infrared spectroscopy (NIRS) analyzer (Perten Instruments, Hägersten, Sweden). The equipment was calibrated using standard chemical analysis methods (Soxhlet extraction for oil and Kjeldahl method for protein) to ensure data accuracy. Analysis of variance (ANOVA) was performed using SAS 9.4 software, and broad-sense heritability (*H*^2^) was calculated.

### 4.2. Genotyping and Population Genetic Analysis

For genotyping, the “Zhongdouxin No. 1” SNP array was used to genotype the newly introduced Uruguayan germplasm and the exported germplasm. The genotyping success rate was 95.95%, yielding an average of 158,327 variant loci. Given the relatively slow decay of linkage disequilibrium (LD) in the soybean genome, the high marker density provided by this array (average spacing < 10 kb) offered extremely high genomic resolution for the 98 core accessions. This allowed for the precise detection of fine-scale selection signatures across the whole genome and the accurate anchoring of key candidate genes, ensuring the statistical power of the genetic analysis.

PLINK 1.9 was used to filter low-quality markers (MAF < 0.05, GENO > 0.1). Population structure analysis was conducted using PCA. VCFtools was used to calculate the genetic differentiation index (*F_ST_*) and nucleotide diversity (*pi*) between populations, scanning for whole-genome selection signals with a 100-kb window and a 10-kb step size.

### 4.3. Haplotype and Favorable Allele Analysis

Despite the moderate sample size, the high heritability and distinct population structure allowed for the effective detection of major selection signatures. Based on the array data, SNPs within key candidate gene regions (e.g., *GmDGAT1*, *GmbZIP123*) were extracted. Combined with a list of 42 oil-related genes compiled from the published literature (Supplementary Table S1), the total number of “high-oil” favorable alleles carried by each germplasm accession was counted. Differences between populations were compared using *t*-tests.

## 5. Conclusions

This study confirms that Uruguayan soybean germplasm represents a specialized, high-oil gene pool shaped by intense directional selection, distinct from the center of origin in China. We identified GmDGAT1 and GmbZIP123 as critical targets where favorable haplotypes have been fixed in Uruguayan lines but remain variable in Chinese germplasm. These findings imply that the oil content gap between the two populations is driven by specific, manipulable genetic factors. Consequently, we propose that Uruguayan germplasm should be utilized as a reservoir of ‘pre-assembled’ high-oil genetic modules. Future breeding must advance beyond simple hybridization to focus on the precise introgression of these modules into broadly adaptive Chinese backgrounds, utilizing strategies such as marker-assisted selection or gene editing. This strategy offers a viable roadmap to break the negative correlation between protein and oil, ultimately securing higher vegetable oil production efficiency.

## Figures and Tables

**Table 1:** Descriptive statistics of seed protein and oil content in three soybean populations.

Group	Protein Mean	Protein SD	Protein Variance	Oil Mean	Oil SD	Oil Variance
CN	43.61%	1.80	3.23	19.42%	1.05	1.11
UY_N	40.24%	1.18	1.39	21.56%	0.55	0.30
UY_T	36.30%	2.14	4.60	21.48%	1.03	1.07

**Table 2:** Variance and broad-sense heritability of oil content in Chinese and Uruguayan soybean germplasm.

Evaluation Group	Number of Accessions	Number of Environments	*MS_G_*	*MS_RES_*	*H* ^2^	*95% Cl*
CN	48	5	5.56	0.75	0.87	0.79–0.92
UY_N	24	7	2.12	0.46	0.82	0.62–0.89
UY_T	26	7	7.48	0.14	0.98	0.97–0.99
Combined	72	7	11.97	0.90	0.92	0.89–0.95

Note: *MS_G_*, genotypic mean square; *MS_R__ES_*, residual mean square; *H*^2^, broad-sense heritability. The variance component method was used for calculation: *H*^2^ = (*MS_G_* − *MS_RES_*)/*MS_G_*. Values in parentheses indicate the 95% confidence intervals for *H*^2^.

**Table 3:** List of candidate genes associated with oil content identified within or near the selective sweeps.

Gene Name	Chr	Position	Gene ID (Wm82)	*F_ST_* (Nearest SNP)	*F_ST_* (Region Peak)	Putative Function
*GmBloom1*	13	35,163,354	Glyma.13G241700	0.13	0.3	Seed blooming and oil content
*GmDGAT1*	17	4,054,733–4,058,120	Glyma.17G053300	0.00	0.51	TAG biosynthesis
*GmbZIP123*	6	801,327–804,550	Glyma.06G010200	0.68	0.77	bZIP transcription factor
*GmST05*	5	41,853,287	Glyma.05G244100	0.69	0.69	Phosphatidylethanolamine-binding protein
*GmDREBL*	12	9,187,362	Glyma.12G103100	0.57	0.66	DREB-type transcription factor
*GmZF351*	6	47,897,987	Glyma.06G290100	0.02	0.46	Zinc finger C-x8-C-x5-C-x3-H type
*GmLEC2a*	20	5,023,173–5,023,099	Glyma.20G035800	0.32	0.32	AP2/B3-like transcriptional factor
*GmRab5a2*	13	26,781,452	Glyma.13G153000	0.36	0.36	Small GTPase
*GmSWEET39*	15	3,875,093–3,876,757	Glyma.15G049200	0.04	0.23	Sugar efflux transporter

**Table 4:** Frequency distribution of *GmDGAT1* and *GmbZIP123* haplotypes across different soybean germplasm groups.

Type	*GmDGAT1* Haplotype			*GmbZIP123* Haplotype				
	Hap 1	Hap 2	Hap 3	Hap 4	Hap 5	Hap 6	Oil Content%	Number
CN	0.257	0.650	0.093	0.170	0.750	0.080	19.42	48
UY_N	0.783	0.150	0.067	1.000	0.000	0.000	21.56	24
UY_T	1.000	0.000	0.000	0.880	0.050	0.070	21.41	26

Note: CN, Chinese germplasm; UY_N, Uruguayan conventional lines; UY_T, Uruguayan transgenic lines. The values indicate the proportion (frequency) of accessions carrying a specific haplotype within each sub-population.

**Table 5:** Haplotype characterization and nucleotide variation patterns of candidate genes *DGAT1* and *GmZIP123*.

	*GmDGAT1*					*GmbZIP123*			
Haplotype	Gm17 4054733	Gm17 4056210	Gm17 4058100	Mutation Pattern	Haplotype	Gm06 19602265	Gm06 19622418	Gm06 19676661	Mutation Pattern
Hap1	C	A	T	C-A-T	Hap4	G	T	C	G-T-C
Hap2	G	G	T	G-G-T	Hap5	T	C	T	T-C-T
Hap3	G	G	C	G-G-C	Hap6	T	T	C	T-T-C

Note: The SNP markers are named according to their chromosome and physical position (bp) based on the *Glycine max* reference genome (Wm82.a2.v1). *DGAT1* is located on Chromosome 17, and *GmZIP123* is located on Chromosome 6. The alleles are represented by A (Adenine), T (Thymine), C (Cytosine), and G (Guanine). Hap1 represents the predominant haplotype with the highest frequency in the studied population.

## Data Availability

The data presented in this study are available in the article and its Supplementary Materials.

## Supplementary Materials

The following supporting information can be downloaded at: https://www.mdpi.com/article/10.3390/plants15050800/s1, Table S1: 42 known oil-related gene loci.
